# Iron Metabolism Genes Shape the Course of Liver Fibrosis in Chronic Hepatitis C: From Disease Progression to Reversal After Direct-Acting Antivirals Treatment

**DOI:** 10.3390/v17101302

**Published:** 2025-09-26

**Authors:** Joana Ferreira, Manuel Bicho, Paula Faustino, Fátima Serejo

**Affiliations:** 1 Institute for Scientific Research Bento Rocha Cabral, 1250-047 Lisbon, Portugal; manuelbicho@medicina.ulisboa.pt; 2 Genetics Laboratory, Environmental Health Institute (ISAMB), Associated Laboratory TERRA, Lisbon Medical School, University of Lisbon, 1649-028 Lisbon; Higher Institute of Agronomy, 1349-017 Lisbon, Portugal; paula.faustino@insa.min-saude.pt (P.F.); fatimaserejo53@gmail.com (F.S.); 3 Human Genetics Department, National Institute of Health Dr. Ricardo Jorge, 1649-016 Lisbon, Portugal; 4 Gastroenterology and Hepatology Department, Hospital de Santa Maria, 1649-028 Lisbon, Portugal

**Keywords:** Chronic hepatitis C, fibrosis, iron metabolism, oxidative stress, genetic polymorphism, direct-acting antivirals

## Abstract

Chronic hepatitis C (CHC) is linked to iron overload, which significantly correlates with liver fibrosis. This study aimed to assess whether genetic polymorphisms related to iron metabolism are associated with fibrosis severity, predict improvement in fibrosis after HCV clearance with direct-acting antivirals (DAAs) and influence iron-related metabolic markers before treatment. A total of 329 CHC patients were included, 134 of whom received DAAs therapy. Liver fibrosis was assessed using transient elastography (FibroScan), and biochemical parameters were measured using standard methods. Eighteen genetic polymorphisms within five iron metabolism-related genes were analyzed using PCR-RFLP, endpoint genotyping, or next-generation sequencing (NGS). Before DAA treatment, patients with severe fibrosis showed higher levels of serum iron (Fe), total iron-binding capacity (TIBC), and ferritin (Ft). SLC40A1 rs1439816_GG was associated with an increased risk of severe fibrosis compared with GC or CC genotypes. SLC40A1 rs11568351_GC genotype was linked to a higher likelihood of remaining cirrhotic after HCV clearance. Elevated iron parameters were observed in carriers HFE C282Y_CY, TF IVS 11 G>A, and BMP2 570 A>T. Overall, polymorphisms in iron metabolism genes may influence both the severity of liver fibrosis prior to treatment, its regression after DAA therapy and the regulation of iron metabolism in CHC patients.

## 1. Introduction

Iron is an essential nutrient for humans and plays a vital role in cellular homeostasis, as it participates in numerous cellular processes [1,2].

However, iron can also be biochemically harmful because, in excess, it promotes the synthesis of toxic reactive oxygen species that damage cell membranes, proteins, lipids, and DNA, ultimately leading to a process called ferroptosis [2,3]. Under iron overload conditions, the liver is the primary target organ. High hepatic iron concentrations can result in hepatocellular injury, fibrosis, and cirrhosis [4].

All steps necessary to maintain iron homeostasis are regulated at both systemic and cellular levels [5].

The liver is the main iron storage organ. It plays an essential role in iron metabolism, as it synthesizes transferrin (Tf), the main carrier protein in plasma, and ferritin (Ft), the primary storage protein [6]. Hepcidin is also produced predominantly by hepatocytes making hepatocytes the main source of this key systemic iron-regulatory hormone. Hepcidin plays a central role in iron metabolism by binding to the iron exporter ferroportin (FPN) to regulate intestinal iron absorption and iron release from storage sites. By controlling FPN degradation in enterocytes, macrophages, and hepatocytes, hepcidin ensures iron homeostasis and prevents both overload and deficiency [7].

Chronic hepatitis C (CHC) is associated with iron overload in 10–42% of individuals. Moreover, 20–35% of CHC patients show increased levels of transferrin saturation (TS), serum iron (Fe), and ferritin (Ft), and these parameters correlate significantly with liver fibrosis [1,8].

In chronic viral hepatitis, iron accumulation is thought to result mainly from hepatocellular injury, whereby infected hepatocytes release hemosiderin that is subsequently taken up by Kupffer cells (KCs). Additional mechanisms may also contribute, such as hepatocyte regeneration, cytokine release, and, such as hepatocyte regeneration, cytokine release, and altered iron uptake due to chronic necroinflammation and intrahepatic shunting [9].

Inflammatory conditions are often associated with significant changes in systemic iron metabolism. The main factor responsible for these changes is the increased expression of hepcidin, which controls intestinal iron absorption and release from macrophages [10,11]. Moreover, hepatitis C virus (HCV) itself influences iron absorption by the oxidative stress-mediated negative regulation of hepcidin expression [1].

**Figure 1:**
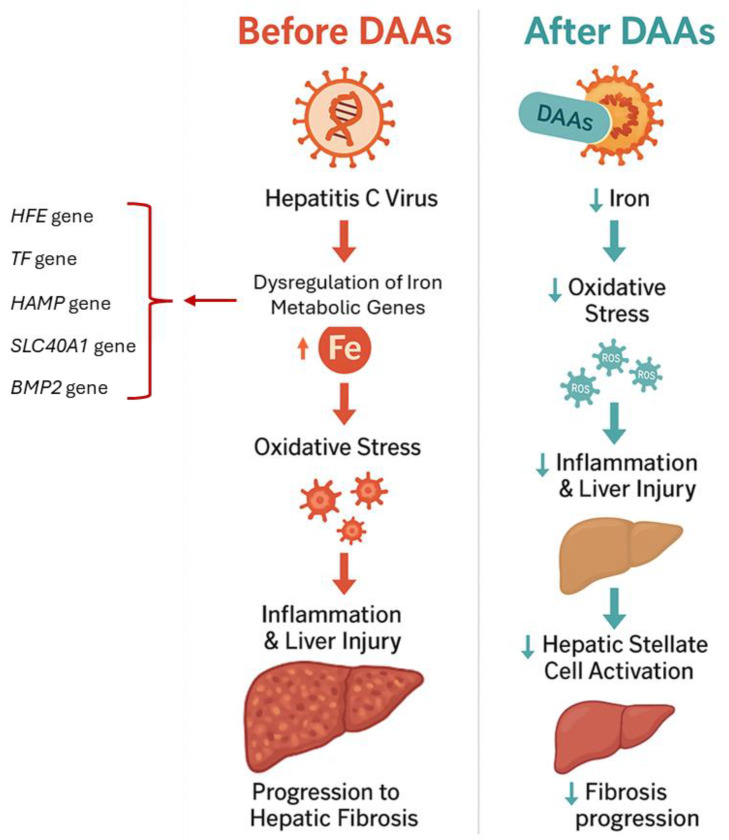
Consequences of HCV on iron accumulation, oxidative stress and hepatic fibrosis, and its reversible effects after DAAs.

HCV clearance with direct-acting antivirals (DAAs) normalizes serum iron metabolism parameters and is expected to restore hepcidin expression. Although normalization may occur without additional interventions, pharmacological targeting of the hepcidin/ferroportin axis has emerged as a potential adjuvant therapy to correct iron trafficking. Abnormalities of iron metabolism in CHC are either a direct effect of the virus or a consequence of viral infection, such as inflammation, which is resolved with HCV elimination [12,13] (Figure 1).

The HFE protein is a class I human leukocyte antigen (HLA) that is expressed on the cell membrane in association with β2-microglobulin (β2M) and has iron transport properties [14,15,16]. It is codified by the *HFE* gene located on the short arm of chromosome 6 (6p22.1). This gene consists of seven exons, only the first six of which encode the six domains of the HFE protein (signal peptide, α1, α2, α3, transmembrane, and cytoplasmic).

There are two frequent mutations in the *HFE* gene. The first, *HFE*: c.187 C>G (rs1799945), located in exon 2, corresponds to a cytosine-to-guanine substitution at position 187, replacing histidine with aspartate at position 63 in the protein (H63D). As this alteration occurs in the interaction domain with transferrin receptor 1 (TfR1), it can cause a slight loss of function and, consequently, iron overload [14,17]. The other one, *HFE*: c.845 G>A (rs1800562), located in exon 4, corresponds to a guanine-to-adenine substitution at position 845, leading to the replacement of cysteine by tyrosine at position 282 (C282Y). This alteration changes the conformation of the protein, causing it. This alteration changes the conformation of the protein, causing it to be retained and degraded in the endoplasmic reticulum, thereby preventing its expression on the hepatocyte surface. Its interaction with transferrin receptors 1 and 2 (TfR1 and TfR2) is compromised, leading to a significant iron overload [14,16,17].

Hereditary Hemochromatosis (HH) is a genetic disorder most commonly due to homozygosity for the *HFE* C282Y mutation or compound heterozygosity C282Y/H63D. In this condition, intestinal iron absorption is inappropriately increased, and the excess of iron accumulates in diverse organs, especially in the liver, where they might be responsible for liver damage, and eventually fibrosis, cirrhosis, and carcinoma hepatocellular. However, the role of these *HFE* mutations in fibrosis progression of HCV patients remains controversial [18,19,20,21,22].

Transferrin (Tf) is a protein that transports iron in plasma and facilitates its internalization by target cells enabling its participation in numerous cellular functions. Once in the cytoplasm, Fe^2+^ is oxidized to Fe^3+^ by hephaestin (or ceruloplasmin, in the liver) and incorporated into Tf, which has a high affinity for the ferric form [5]. Tf is found in plasma in three states: apo-transferrin (no iron bound), monoferric transferrin (Tf-Fe^3+^, binding a single iron atom), and diferric transferrin (Tf-(Fe^3+^)_2_ binding two iron atoms). These states enable adaptation to sudden increases in iron absorption and prevent the toxic effects of iron overload [23,24,25].

Tf is encoded by the *TF gene* located on the long arm of chromosome 3 (3q22.1) and consists of 24 exons. A polymorphism within intron 11, *TF*: c.−427A>G (rs3811647), was found to modulate Tf expression [26]. Several studies have suggested a role for serum Tf levels in liver fibrosis progression, reporting lower levels of this protein in patients with advanced fibrosis or cirrhosis [27,28].

Hepcidin is crucial in iron metabolism as a fundamental regulator of iron homeostasis [17]. It degrades ferroportin (FPN1) whenever iron export is not required. Thus, a low level of hepcidin allows FPN1 activity regardless of systemic iron status, leading to excessive iron export into plasma and severe iron overload [16]. Hepcidin is upregulated in inflammation by interleukin 6 (IL-6) and other cytokines through the activator of transcription (JAK/STAT) signaling pathway [29,30,31].

Low hepcidin levels can induce iron overload and oxidative stress, which may result in hepatic injury and fibrosis. On the other hand, as hepcidin can protect the liver by inactivating hepatic stellate cells, lack of this hepcidin-induced suppression results in their activation and the deposition of scar tissue and liver fibrosis [30].

Hepcidin is encoded by the *HAMP* (hepcidin antimicrobial peptide) gene located on the long arm of chromosome 19 (19q13.12). This gene consists of three exons and encodes the hormone hepcidin [32]. It is mainly expressed in the liver in situations of iron overload. Its expression is upregulated via the bone morphogenetic protein (BMP) signaling pathway, mediated by hemojuvelin, transferrin receptor 2, and the HFE protein [29].

*HAMP*: c.-582 A>G polymorphism (rs10421768) is in the *HAMP* gene promoter and is characterized by an adenine-to-guanine substitution, which disrupts the E-box consensus sequence and prevents transcription factor binding [33]. It reduces HAMP transcriptional activity in HepG2 hepatocellular carcinoma cells [29]. Another polymorphism in the promoter of the *HAMP* gene, *HAMP*: c.-1010 C>T (rs10414846), is characterized by a cytosine-to-thymine substitution. One study reported this variant to be in linkage disequilibrium with *HAMP* -582 A>G, giving rise to two promoter haplotypes (CA and TG). In this study, individuals carrying *HFE* H63D and at least one mutated allele of each *HAMP* promoter polymorphism presented higher ferritin levels [34].

BMPs are multi-functional growth factors that belong to the transforming growth factor β (TGF-) superfamily. They have several functions, including regulating cellular homeostasis processes such as proliferation, differentiation, apoptosis, and extracellular matrix remodeling. They are essential in several diseases and pathological conditions like fibrosis [35].

BMPs are involved in the hemojuvelin-bone morphogenetic protein (HJV-BMP) pathway, one of the pathways involved in regulating hepcidin synthesis. The HJV protein acts as a co-receptor for BMPs on the cell surface, and the binding of BMPs to HJV activates the SMAD signaling pathway, inducing the expression of the hepcidin gene [36]. Bone morphogenetic protein 2 (BMP2) is one of the BMPs identified as activators of *HAMP* expression [5]. Moreover, a study from 2018 demonstrated that BMP2 performs an inhibitory effect on hepatic fibrosis through the inhibition of TGF-β1/Smad3 pathways, hepatic stellate cells (HSCs) activation, and epithelial-to-mesenchymal transition (EMT) suppression [37]. On the other hand, *BMP2* gene expression may be modulated by genetic polymorphisms within the *BMP2* gene. Therefore, given the indirect influence of BMPs on iron metabolism and its importance in iron homeostasis, it seems relevant to understand whether a particular variant of the *BMP2* gene, in this case, the *BMP2* 570 A>T (rs235768) polymorphism, is a susceptibility factor for the development of iron overload and for liver fibrosis.

The *BMP2* gene is located on the short arm of chromosome 20 (20q12.3) and consists of 3 exons. The *BMP2*: c.570A>T polymorphism is in exon three and corresponds to an adenine-to-thymine substitution at position 570, leading to an arginine-to-serine change (Arg190Ser) in the pro-peptide region of the protein [38,39].

Ferroportin (FPN1) is a transmembrane iron transporter expressed in macrophages, enterocytes, and hepatocytes. In iron overload, its binding to hepcidin promotes its internalization and degradation, thereby decreasing iron absorption and reducing serum iron levels [32].

FPN1 is encoded by the *SLC40A1* gene, located on chromosome 2q32.2, which comprises 8 exons. Mutations in this gene can lead to either loss or gain of FPN1 function [40,41,42].

The primary objectives of the study were to evaluate in CHC patients the association between iron metabolism-related polymorphisms and liver fibrosis stage, as assessed by transient elastography (TE), and to determine whether they could predict fibrosis regression after HCV clearance with DAAs. We also examined whether these polymorphisms modulate baseline metabolic parameters of iron metabolism associated with severe fibrosis before DAA treatment.

## 2. Materials and Methods

### 2.1. Populations

A group of 329 patients with CHC were prospectively studied. From these, 134 were also evaluated 24 weeks after being treated with Sofosbuvir/Velpatasvir 1 pill per day for 12 weeks, a pangenotypic DAA, and with a sustained response (HCV-RNA undetected 6 months after viral load 0 UI/mL according to European Association for the Study of Liver (EASL) guidelines) [43]. Table 1 summarizes the main characteristics of the studied populations. The two columns before treatment refer to all populations (*n* = 329) and those treated (*n* = 134). Patients were selected, examined, adequately informed, and consented following the WMA Helsinki Declaration [44].

Inclusion criteria (before treatment): presence of positive RNA and anti-HCV for more than six months; age 18 years or older and informed consent. Inclusion criteria (after treatment): sustained response (HCV-RNA undetected 6 months after viral load 0 UI/mL according to European Association for the Study of Liver (EASL) guidelines) [43]; age 18 years or older and informed consent.

Exclusion criteria: other chronic liver diseases (viral hepatitis A and/or B, autoimmune diseases, and other genetic and/or metabolic diseases); concurrent infection with HIV; alcohol consumption > 40 gr/day; pregnant women and individuals with impaired intellectual capacity.

### 2.2. Liver Fibrosis Evaluation:

Liver stiffness (LS) was evaluated by TE using a FibroScan^®^ device (Echosens, Paris, France) with a 5 MHz ultrasound transducer mounted on the axis of a vibrator. The vibrator generates a painless vibration (frequency 50 Hz and amplitude 2 mm) like a “flick”, generating a shear wave propagating through the skin and the subcutaneous tissue into the liver. The velocity of the wave is directly related to the LS. The median value of 10 successful acquisitions was expressed in kilopascals (kPa), with a success rate of at least 60% and an interquartile range (IQR) lower than 30%. LS cut-off values validated in the Gastroenterology and Hepatology Department, Hospital de Santa Maria, Lisbon, Portugal (liver biopsy analysis of 110 patients, Scheuer classification) were used to establish fibrosis stages: 5.43 kPa for F ≥ 2 (PPV 0.78; NPV 0.67); 8.18 kPa for F ≥ 3 (PPV 0.95 NPV 0.93); 12 kPa for F = 4 (PPV 0.93; NPV 0.93). The average value was ≤4.9 kPa [45].

### 2.3. HCV-RNA

Serum HCV-RNA was evaluated by Real-Time PCR Taqman from Roche Diagnostics (test sensitivity < 15 IU/mL).

### 2.4. Metabolic Evaluation

Metabolic parameters of iron metabolism were evaluated before antiviral treatment using standard methods from the Clinic Pathology Department of the hospital (reference values described): free iron (Fe ≤ 170 μg/dL), transferrin saturation (TS ≤ 40%), total iron binding capacity (TIBC ≤ 450 μg/dL), and ferritin (Ft ≤ 291 ng/mL).

### 2.5. DNA Extraction

Peripheral blood was collected in EDTA and stored at −20 °C until analysis. DNA was isolated from leukocytes by an adapted non-enzymatic DNA extraction procedure [46].

### 2.6. Genetic Polymorphisms Identification

In the *HFE* gene, two common polymorphisms, *HFE* C282Y (845 G>A; rs1800562) and HFE H63D (187C>G; rs1799945) were evaluated by multiplex PCR-ARMS (polymerase chain reaction amplification refractory mutation system), whose assay conditions and primers sequences were described by Baty D et al. [47]. In each experiment, in addition to the patients’ DNA to be analyzed, the appropriated positive DNA controls as well as one PCR negative control were added. For each patient, we performed two reactions of PCR-ARMS (Mix 1 and 2), whose amplified products were run side by side in a 2% agarose gel for 40 min at 70 volts. Table 2 and Table 3 describe primers and all the reagents and amplification conditions, respectively.

In the Tranferrin gene (*TF*), one polymorphism located within intron 11, IVS 11 G>A (rs3811647), was studied by PCR followed by Sanger sequencing. Primers (designed in house using NCBI Primer-BLAST), reagents, and amplification conditions were optimized and are described in Table 4 and Table 5. Primers (designed in house using NCBI Primer-BLAST), reagents, and amplification conditions were optimized and are described in Table 4 and Table 5. The appropriated wild-type, heterozygous, and homozygous controls were included, along with a negative PCR control. Then, the amplification success was confirmed in a 2% agarose gel for 40 min at 70 volts, and the PCR products were purified using the ExoSAP-IT™ PCR Product Cleanup kit (Applied Biosystems, Foster City, CA, USA) and sequenced using the Big Dye © Terminator V1.1 Cycle Sequencing Kit (Applied Biosystems, Foster City, CA, USA).

Polymorphisms within *SLC40A1*, *HAMP*, and *BMP2* genes were studied by PCR followed by NGS (Next Generation Sequencing). For this process to happen, five long PCRs were performed, totaling approximately 11 kb of DNA amplified per individual. A portion of 1139 bp containing the *HAMP* gene promoter was amplified for the study of the -1010 C>T (rs10414846) and -582 A>G (rs10421768) polymorphisms. Exon 3 of the BMP2 gene was amplified in a 1031 bp fragment to identify the *BMP2* 570 A>T (rs235768) polymorphism. Concerning the SLC40A1 gene, the entire gene was amplified in three fragments: frag. 1—from the 5′-UTR region to part of intron 2 (991 bp); frag. 2—from intron 2 to part of intron 5 (3747 bp); frag. 3—from intron 5 to the 3′-UTR region (4175 bp). Primers, reagents, and amplification conditions were optimized and are described in Table 6 and Table 7. Amplification success for each fragment was confirmed in 1% agarose gels for 40 min at 70 volts. Based on the intensity of the PCR products’ bands, a pool of the five amplified fragments for each patient was made and the NGS technique was performed. This process was preceded by amplicon pool purification with the Agencourt AMPure XP PCR Purification kit (Beckman Coulter, Brea, CA, USA) according to manufacturer’s instruction, followed by quantification in a Qubit^®^ 3.0 fluorometer (Life Technologies, Carlsbad, CA, USA). The first step of NGS included sequencing library preparation using the Nextera XT DNA Library Prep (Illumina, San Diego, CA, USA). Then, libraries were sequenced in MiSeq equipment (Illumina, San Diego, CA, USA) using a 0.5 Gb flow cell. During sequencing, the equipment carried out a primary quality control analysis. Data analyses of the sequencing results comprised three steps: quality control using the MultiQC^®^ 1.6.dev0 software; mapping reads to reference genome GRCh38 using bowtie; and variant calling where base call quality values were corrected for systematic error with the software GATK^®^. Furthermore, varying positions were filtered for a variant quality < 130 (Phred scale) and sample genotypes were only considered when there was a minimum read depth of 20x and a genotype quality < 90. To accept genotyped heterozygous positions, the allelic depth reads were also taken into consideration to account for allelic unbalance, genotypes with an allelic balance below 30 % were excluded (0.3 ≤ accepted allelic balance ≤ 70). After NGS data analysis, 47 variants in the SLC40A1 gene were identified. However, our study only considered those with a minor allele frequency equal to or superior to 1% (polymorphisms) totalizing 11 variants.

**Statistical analysis:** Statistical analysis was performed using SPSS 24.0 for Windows. Data were inserted into a database built in this same program, safeguarding the confidentiality of the participants’ identities. Metabolic parameters were treated as continuous variables. Two groups were established for the analysis of liver fibrosis. Patients with mild and moderate fibrosis (F1 and F2; F1/2) and patients with severe fibrosis and cirrhosis (F3 and F4; F3/4) were grouped so that the frequencies would not be too low in some cases. Descriptive analysis was performed assuming univariate and bivariate analysis. The non-parametric Kolmogorov–Smirnov test first tested the normality for continuous variables. As there was at least one variable with a non-normal distribution, all variables were analyzed using non-parametric tests and were described as mean and 95% confidence interval. Absolute and relative frequencies were used to define the categorical variables. In the bivariate analysis, categorical variables were compared using the Chi-square test or Fisher’s exact test, and the Odds Ratio (OR) was calculated with their respective confidence intervals whenever justifiable. Statistical comparisons were performed for continuous variables using the non-parametric Mann–Whitney and Kruskal–Wallis tests. We use Paired Sample Tests to make comparisons before and after DAA treatment. Whenever the compared groups differed in gender, age, or BMI, statistical analysis was conducted with adjustments for the parameter/s that differed. It was considered a statistically significant result for a *p*-value < 0.05.

## 3. Results

Analyzing Table 1 in the section “Materials and methods”, we can observe that CHC patients are mainly male and have fibrosis stages F1/2. They have a borderline average BMI value and are around 50 years old.

### 3.1. Association of Metabolic Parameters of Iron Metabolism with Fibrosis Stage Before DAAs Treatment

Baseline metabolic parameters of iron metabolism were compared between patients with higher fibrosis stages (F3/4) and those with lower ones (F1/2) before DAAs treatment. Patients with severe fibrosis showed higher levels of Fe, TIBC and Ft (Table 8).

### 3.2. Association of Genetic Polymorphisms Related to Iron Metabolism with Liver Fibrosis Stage

Genotype frequencies of the genetic polymorphisms studied were compared between patients with higher fibrosis stages (F3/4) and those with lower ones (F1/2) before DAAs treatment.

Before DAAs treatment, patients with GG genotype of *SLC40A1* rs1439816 have 3.937 risk for presenting severe fibrosis stages (F3/4) compared with carriers of the C allele (genotypes GC or CC) (Table 9).

### 3.3. Association of Genetic Polymorphisms Related to Iron Metabolism with the Improvement of Liver Fibrosis After HCV Clearance with DAAs

Genotype frequencies of the genetic polymorphisms studied were compared between patients who improved liver fibrosis (F3/4 to F1/2) with those who maintained F3/4. No significant results were found. We did the same comparison study between patients with regression of liver fibrosis from F4 (cirrhosis) to F1, 2, or 3 after treatment and those who stayed cirrhotic (F4), and we obtained significant results. However, due to the low number of patients we did not consider this result as valid.

Patients with GC genotype of *SLC40A1* rs11568351 have 11.429 risk of staying cirrhotic (F4) after DAAs treatment (Table 10) (only significant results are shown).

### 3.4. Association of Genetic Polymorphisms with Metabolic Parameters of Iron Metabolism Associated with Severe Liver Fibrosis After HCV Clearance with DAAs

As shown in Table 8, patients with severe fibrosis before DAAs treatment showed higher baseline values of Fe, TIBC, and Ft.

Baseline Fe, TIBC, and Ft were compared between the different genotypes of all the studied genetic polymorphisms of iron metabolism. Co-dominant, dominant, and recessive genetic models were applied. For the two last, only significant results are shown.

Higher values of Fe were found in heterozygous CY for *HFE* C282Y (*p* = 0.009, *p* = 0.005, respectively), TIBC was higher in patients carrying allele A (genotypes GA or AA) of *TF* IVS 11 G>A (*p* = 0.011), and Ft was higher in patients heterozygous CY for *HFE* C282Y (*p* = 0.005) and in those carrying allele T (TT or TA) of *BMP2* 570 A>T (*p* = 0.033) (Table 11).

## 4. Discussion

In this study, we observed that our CHC patients exhibited elevated levels of iron metabolism biomarkers (Fe, TIBC, and Ft), associated with advanced stages of liver fibrosis before DAA treatment, partially consistent with the findings of Guyader et al. and Vagu et al. [6,8,48]. In a situation of liver inflammation, iron may be released from damaged hepatocytes [48]. Some studies suggest that high iron levels exacerbate necroinflammatory activity in CHC and accelerate fibrosis progression [49,50]. Moreover, iron accumulation in CHC causes oxidative stress and consequent liver damage, promoting hepatocyte necrosis/apoptosis, activation of hepatic stellate cells, and fibrogenesis through actin and collagen proliferation [51].

Regarding Ft, we obtained the same results as Vagu et al. [6]; serum Fe levels are essential in determining the severity of liver disease associated with liver fibrosis and necroinflammatory activity [6]. As with Fe, Ft is released by damaged hepatocytes under inflammatory conditions, and the greater the inflammation, the more Fe is released [48]. The same study reported lower levels of TIBC associated with more severe fibrosis stages. This result contradicts our findings, as we observed higher mean TIBC values in patients with advanced fibrosis. We did not identify other studies that support this result.

In the presence of HCV infection, hepcidin transcription is suppressed, causing a decrease in its expression, which increases iron export via ferroportin in enterocytes and macrophages, thereby enhancing duodenal iron absorption and iron release from macrophages [51]. Moreover, the increase in hepatic iron in viral hepatitis may be due to a defensive process whereby liver cells accumulate iron to limit pathogen access and inhibit proliferation [52]. With HCV elimination, these two processes are reversed, leading to a decrease in the values of iron parameters. This result is in concordance with the study from Hasan Y. et al. [12]. who reported normalization of serum iron parameters shortly after HCV clearance with DAAs, suggesting a direct viral effect on iron metabolism. They also demonstrated that this normalization has lasting effects, indicating that CHC-related iron abnormalities may stem from virus-induced inflammation that resolves with viral elimination [12].

The interplay between oxidative stress and endoplasmic reticulum (ER) stress is central to HCV pathogenesis. Liver-focused reviews place HCV within a broader oxidative framework: virus-driven ROS promotes fibrosis progression and carcinogenesis, with redox–TGF-β crosstalk and mitochondrial injury acting as key mediators [53]. The HCV core protein disrupts mitochondrial electron transport, increasing reactive oxygen species (ROS), depleting glutathione, and promoting mitochondrial permeability transition [54,55]. In parallel, structural proteins such as core, E1, and E2 accumulate in the ER, where they trigger the unfolded protein response (UPR), upregulate GRP78 (8 kDa glucose-regulated protein) and GADD153/CHOP (Growth arrest and DNA damage-inducible protein 153), and downregulate Bcl-2 (B-cell lymphoma 2), sensitizing hepatocytes to apoptosis and linking ER dysfunction to oxidative imbalance [54,56]. The combined disruption of mitochondrial respiration and ER protein folding establishes a reinforcing cycle of stress signaling that compromises hepatocyte viability. These stress pathways further affect proteasome regulation, inflammation, and fibrogenesis. Proteasome activity, enhanced under mild oxidative stress, is suppressed under exacerbated imbalance, such as during ethanol exposure, impairing antigen presentation and interferon signaling [57]. Nonstructural protein 5A (NS5A) modulates this crosstalk by sustaining GRP78 expression, suppressing ER stress-induced apoptosis via inhibition of caspase activation and Bax (Bcl-2-associated X protein) expression and upregulating anti-apoptotic proteins XIAP (X-linked inhibitor of apoptosis protein) and c-FLIP (cellular FLICE-like inhibitory protein) [58]. NS5A also promotes calcium-dependent mitochondrial ROS production and activates NF-κB (Nuclear factor kappa-light-chain-enhancer of activated B cells) and STAT3 (Signal transducer and activator of transcription 3), linking stress responses to pro-survival and oncogenic signaling [58].

HCV pathogenesis is increasingly framed as a bidirectional loop between oxidative stress and ER stress that sustains viral persistence and liver injury. HCV infection activates ER-stress signaling, which remodels metabolism and innate immunity, while simultaneously amplifying mitochondrial dysfunction, ROS generation and induce pro-fibrogenic mediators such as TGF-β1/β2 and hepcidin, connecting these pathways to fibrosis and hepatocarcinogenesis [59]. Together, these findings highlight how reciprocal amplification of oxidative and ER stress drives hepatocyte injury, chronic inflammation, and malignant transformation, underscoring stress-response pathways as key therapeutic targets.

Regarding the primary objectives of the study, an association was found between genetic polymorphisms of *SLC40A1* with liver fibrosis and its improvement after HCV clearance with DAAs.

CHC patients carrying the homozygous wild-type genotype *SLC40A1* rs1439816 (GG) had an increased risk of severe fibrosis (F3/4) before DAA treatment compared with carriers of the C allele (GC or CC). On the other hand, patients presenting polymorphism in *SLC40A1* rs11568351 (GC) had a significantly higher risk of remaining cirrhotic (F4) after HCV elimination with DAAs. This result should be confirmed by evaluating more patients.

FPN1 is a transmembrane protein that transports iron from the inside of a cell to the outside [60]. By the action of hepcidin on FPN1, serum iron levels are tightly regulated through hepcidin-induced internalization and degradation of FPN1, which controls absorption and recycling [61]. Being the only known iron exporter, its role is crucial for iron homeostasis, and any change in its amount or function can disrupt iron balance within and outside cells.

Mutations in the *SLC40A1* gene that cause loss of function are generally located in sites of interlobular interaction, affecting the formation of the intracellular gate and reducing its activity. They can also compromise the arrival of FPN1 at the plasma membrane, reducing iron export and causing intracellular iron retention. On the other hand, mutations that lead to a gain of a function affect the hepcidin-binding site, and, as a result, FPN is not de-graded. There is a continuous export of iron, an excessive accumulation of iron in the liver, and an iron deficiency in macrophages [40,41,42]. Downregulation of FPN1 in hepatocytes, resulting from iron overload promotes hepatocyte proliferation and macrophage polarization toward the M2 phenotype, leading to secretion of profibrotic factors, activation of myofibroblasts, extracellular matrix deposition, and fibrosis development [62]. Furthermore, the redox-sensitive transcription factor Nrf2 regulates iron efflux from macrophages by promoting FPN1 gene transcription, suggesting that Nrf2 may play a role in controlling iron metabolism during inflammation [63].

No study has reported an association between *SLC40A1* polymorphisms and liver fibrosis. However, the only study linking this gene to metabolic parameters found lower serum iron in homozygous GG carriers, suggesting a protective role of the rs1439816 G allele against iron accumulation [64]. This contrasts with our findings, where the GG genotype was associated with advanced fibrosis, underscoring the need for studies with larger cohorts. Concerning the variant rs11568351, our study revealed a risk for cirrhosis after HCV clearance associated with its presence. In fact, SLC40A1: c.-8C>G, located within the 5’UTR, has recently been associated with higher gene expression and increased FPN1 levels] [42,65]. Moreover, after viral clearance, hepcidin levels drop due to reduced inflammation. This decreases FPN1 degradation and consequently increases iron export, enhancing absorption by enterocytes and release from macrophages and hepatocytes [30,44].

A decrease in intracellular iron triggers the IRE–IRP signaling pathway, which post-transcriptionally regulates FPN1 by blocking its translation, thereby increasing intracellular iron and reducing circulating levels [66].

It is known that the polymorphism *SLC40A1*: c.-8C>G is located near the iron-responsive element (IRE) region of the gene potentially altering IRE–IRP signaling [67]. We hypothesize that this variant increases IRP affinity for the IRE, excessively repressing translation, causing iron accumulation in hepatocytes, and promoting hepatic injury [66].

Moreover, in chronic inflammation, like CHC, cytokines like IL-6 and TNF-α may induce epigenetic modifications, including DNA methylation and histone changes, that repress FPN1 expression, contributing to hepatic iron retention, oxidative stress, and liver injury [68].

Regarding the secondary objective, we found that higher baseline values of Fe were found in heterozygous CY for *HFE* C282Y, TIBC was higher in patients carrying allele A (GA or AA) of *TF* IVS 11 G>A and Ft was higher in patients’ heterozygous CY for *HFE* C282Y and in those carrying allele T (TT or TA) of *BMP2* 570 A>T.

HFE is a major histocompatibility complex (MHC) class I–like protein that interacts with TfR1 and TfR2 and plays a key role in iron homeostasis by regulating hepcidin expression [69]. If the interaction between *HFE* and transferrin receptors is compromised, it can lead to a significant iron overload due to decreased hepcidin expression.

It is known that a specific polymorphism of *HFE*, *HFE* C282Y (845 G>A; rs1800562), alters protein conformation, causing retention and degradation in the endoplasmic reticulum and preventing its expression on the hepatocyte surface [14,16,17]. As such, *HFE* C282Y polymorphism is associated with higher serum iron, attributed to increased iron export from storage cells to plasma transferrin via ferroportin because of decreased hepcidin levels [70].

In our study, at first, we expected CHC patients with the *HFE* mutant allele (C282Y) to have higher iron overload and, ultimately, severe fibrosis stages. In fact, we observed significant higher values of Fe and Ft in this group of CHC patients heterozygous for that mutant allele (Table 11). However, we did not find any association with the fibrosis stage. There may be several reasons for this fact, for example, because HH is an autosomal recessive disease and our CHC patients are only heterozygous for this C282Y mutation, because the C282Y mutation has low penetrance, and because the manifestations of the disease are influenced by various factors beyond the genotype, such as lifestyle factors. Thus, our results agree with some studies that point to the fact that subjects homozygous or heterozygous for the *HFE* C282Y show increased levels of serum iron, ferritin, and transferrin saturation [71,72,73]. However, another study showed a different result revealing that a significant subgroup of homozygous for *HFE* C282Y had higher values of Ft and that this polymorphism is associated with cirrhosis [74].

In the same way, conflicting results have been published when investigating CHC patients with *HFE* mutations. It was demonstrated that patients with chronic HCV infection and heterozygous for the C282Y or H63D allele, even with minor increases in iron load, had more risk of liver cirrhosis [18,19,20,21]. Conversely, other studies showed that these mutations do not modulate the accumulation of iron or the progression of liver disease in CHC patients [22].

Considering the above and the heterogeneity of published findings, it can be concluded that the *HFE* genotype, in conjunction with other factors, may influence — or not — the progression of fibrosis in patients with CHC as well as in those without it. Further research needs to be conducted to clarify this matter.

Studies from 2009 and 2011 found an association between serum Tf and the polymorphism *TF* IVS 11 G>A (rs3811647) located in intron 11 of the *TF gene*. These results were confirmed in a more recent study from 2021 that showed 21% higher transferrin levels associated with AA carriers, 24% higher unsaturated iron-binding capacity, and 25% lower transferrin saturation compared to GG carriers [75]. Previous studies have shown an association between *TF* IVS 11 G>A polymorphism and metabolic parameters of iron metabolism with increased Tf and TIBC associated with the mutant allele [76,77]. Allele A is associated with an enhancement of protein expression. This allele might create a binding site for the glucocorticoid receptor (GR), which is lost with the presence of allele G. As glucocorticoids influence some iron metabolism genes, GR might regulate glucocorticoid-dependent differences in TF allele expression [26].

Our results are aligned with the above ones as we found an association between this polymorphism and an increase in TIBC.

*BMP2* 570 A>T polymorphism was associated with iron overload in hemochromatosis patients. Moreover, homozygous TT presented higher Ft and TS. Our study came with some of these results as we found higher values of Ft in carriers of allele T. *BMP2* 570 A>T polymorphism is in the pro-peptide region of the protein. This region is essential in BMP maturation as the mature protein requires the formation of heterodimeric complexes with the cleaved pro-peptide for optimal secretion and trafficking within the cell. So, this specific polymorphism of BMP2 may result in defective BMP2 secretion and/or addressing, compromising the SMAD signaling pathway and hepcidin gene expression, increasing the risk of iron overload and fibrosis [38,39].

In our study, the polymorphisms of *HAMP* gene were the only ones that showed no association with fibrosis stage or iron-related metabolic parameters.

Studies in humans and animal models confirmed the relevant role of hepcidin in iron metabolism. A study with genetically modified mice showed that animals with reduced hepcidin expression developed severe iron overload while those with increased expression had severe iron deficiency anemia at birth [78]. In humans, hepcidin overexpression in inflammation causes anemia of chronic diseases characterized by iron-restricted erythropoiesis [79]. On the other hand, the presence of inactivating mutations of hepcidin results in a rare form of juvenile hemochromatosis [80].

As hepcidin is a crucial modulator of systemic iron levels, metabolic changes in iron metabolism may be due to differences in its expression levels, potentially influenced by mutations within the *HAMP* gene promoter. However, the relationship between *HAMP* polymorphisms and iron metabolic changes has yielded conflicting results.

A study from 2009 associated *HAMP* -582 A>G polymorphism with higher liver iron concentration and higher serum Ft levels in beta-thalassemic patients [81]. In 2010, another study reported that the *HAMP* -582 A>G variant decreases *HAMP* expression. However, no significant differences between the genotypes were observed in the Fe, Tf, TS, or Ft levels [29]. In the same year, similar results were obtained by Silva B. et al. reporting that the *HAMP* -582 A>G variant was not associated with elevated Ft levels in healthy subjects [82]. More recently, in 2019 and 2020, Fekri K. et al [83]. and Zarghamian P. et al [84]., supported those results as they found no significant correlation between Ft and *HAMP* -582 A>G polymorphism. However, Zarghamian P. et al [84]. reported significantly greater Fe deposition in cardiac tissue among β-thalassemia patients homozygous for the G allele [83,84].

## 5. Conclusions

The identification of iron-related genetic polymorphisms associated with advanced baseline fibrosis or lack of cirrhosis regression after HCV clearance may have important implications for the advancement of personalized medicine.

These genetic variants could serve as predictive biomarkers, enabling the identification of individuals at higher risk of persistent hepatic fibrosis or cirrhosis despite viral clearance. This may facilitate a more refined risk stratification model, extending beyond viral eradication as the only therapeutic endpoint. On the other hand, surveillance protocols could be tailored based on genetic risk. Patients carrying the risk alleles may benefit from intensified follow-up strategies, including more frequent liver stiffness measurements or imaging for early detection of hepatocellular carcinoma, thereby improving long-term outcomes.

The genetic insight raised by this type of study may also guide targeted therapeutic interventions. First, if the identified polymorphisms are functionally linked to pathways involved in fibrogenesis or hepatic repair, they could represent targets for novel antifibrotic therapies or support individualized selection of adjunctive treatments after SVR. Secondly, the identification of iron-related genetic variants may be used to select post-DAA patients with persistent iron overload and advanced fibrosis or cirrhosis who may benefit from adjuvant interventions such as therapeutic phlebotomy, hepcidin agonists or modulators, antioxidant therapies, or dietary strategies to limit iron intake. It may serve a supportive role in managing residual hepatic injury and reducing long-term complications.

In sum, the integration of host genetic information—particularly polymorphisms associated with higher fibrosis stages and cirrhosis post-HCV clearance—represents a promising step toward precision hepatology, aligning with the broader goals of personalized medicine.

Although this study highlights potential genetic associations of interest, the relatively small sample size limits the statistical power and generalizability of the findings. To robustly confirm these associations and to determine their true clinical significance, further studies are needed in larger populations.

## Figures and Tables

**Table 1:** Characterization of CHC patients before and after DAAs treatment.

	Before DAAs Treatment (*n* = 329)		Before DAAs Treatment (*n* = 134)		After DAAs Treatment (*n* = 134)	
Parameter	Mean	95% CI	Mean	95% CI	Mean	95% CI
**Age (years)**	48.93	[47.57–50.28]	53.42	[51.47–55.36]	53.51	[51.49–55.39]
**BMI (Kg/m^2^)**	25.25	[24.80–25.06]	25.06	[20.78–26.10]		
					25.12	[20.79–25.90]
**HCV-RNA (IU/mL)**	2.16 × 106	[1.51 × 10^6^–2.82 × 10^6^]	2.16 × 10^6^	[2.03 × 10^6^–4.8 × 10^6^]	0.00	-
**Parameter**	***n***	**%**	***n***	**%**	***n***	**%**
**Gender**						
**Female**	124	37.7	58	43.3	58	43.3
**Male**	205	62.3	76	56.7	76	56.7
**Liver fibrosis**						
**F_1_**	84	25.6	29	21.6	59	44.0
**F_2_**	130	39.5	53	39.6	45	33.6
**F_3_**	35	10.6	13	9.7	11	8.2
**F_4_**	80	24.3	39	29.1	19	14.2
95% CI—95% confidence interval for mean; *n*—number of patients; %—percentage of patients; F_1_—mild fibrisis, F_2_—moderate fibrosis; F_3_—severe fibrosis, F_4_—cirrhosis						

**Table 2:** Primers used and amplification fragments obtained in PCR-ARMS for the study of the two *HFE* polymorphisms.

Polymorphism	Primers	Amplification Fragments
***HFE* C282Y** **(rs1800562)**	Forward_Wt: 5′–GCTGATCCAGGCCTGGGTGCTCCACCTGCC–3′ or Forward_Mut: 5′–GCTGATCCAGGCCTGGGTGCTCCACCTGCT–3′ Reverse: 5’–TGGCAAGGGTAAACAGATCC–3′	309 bp
***HFE* H63D** **(rs1799945)**	Forward_Wt: 5′–AGTTCGGGGCTCCACACGGCGACTCTCAAG–3′ or Forward_Mut: 5′–AGTTCGGGGCTCCACACGGCGACTCTCAAC–3′ Reverse: 5’–ACATGGTTAAGGCCTGTTGC–3′	177 bp

**Table 3:** Amplification conditions and reagents used in PCR-ARMS for the study of the two *HFE* polymorphisms.

Polymorphism	Amplification Conditions		Reagents
***HFE* C282Y** **(rs1800562)** ***HFE* H63D** **(rs1799945)**	**1 cycle** Initial denaturation: 94 °C, 4 min **30 cycles** Denaturation: 94 °C, 30 s Annealing: 59 °C, 30 s Extension: 72 °C, 30 s **1 cycle** Final extension: 72 °C, 5 min	**Mix 1**	DNA [25 ng/μL]: 0.5 µL Primer F_C282Y_Wt (25 pmol/μL): 0.5 μL Primer R_C282Y (25 pmol/μL): 0.5 μL Primer F_H63D_Mut (25 pmol/μL): 0.5 μL Primer R_H63D 25 pmol/μL): 0.5 μL dNTP Mixture (2.5 mM each): 0.5 μL * Buffer (10x): 2.5 μL BSA (10 mg/mL): 0.35 μL GoTaq G2 Flexi DNA polymerase (5 U/μL): 0.15 μL H_2_O: 19 μL
***HFE* C282Y** **(rs1800562)** ***HFE* H63D** **(rs1799945)**		**Mix 2**	DNA [25 ng/μL]: 0.5 µL Primer F_C282Y_Mut (25 pmol/μL): 0.5 μL Primer R_C282Y (25 pmol/μL): 0.5 μL Primer F_H63D_Wt (25 pmol/μL): 0.5 μL Primer R_H63D (25 pmol/μL): 0.5 μL dNTP Mixture (2.5 mM each): 0.5 μL * Buffer (10x): 2.5 μL BSA (10 mg/mL): 0.35 μL GoTaq G2 Flexi DNA polymerase (5 U/μL): 0.15 μL H_2_O: 18.5 μL
* Buffer (10x): 166 mM (NH_4_)_2_SO_4_; 670 mM Tris-HCl pH = 8.8; 67 mM MgCl_2_; 0.067 mM EDTA; 100 mM β-mercaptoethanol Primer F_Wt: Primer Forward Wild Type; Primer F_Mut: Primer Forward Mutated; Primer R: Primer Reverse			

**Table 4:** Primers used and amplification fragments obtained in PCR for the study of *TF* polymorphism.

Polymorphism	Primers	Amplification Fragment
***TF* IVS 11 G>A (rs3811647)**	Forward: 5′–TTGCCATGGCTTGCACACAG–3′ Reverse: 5′–TGCCTGTGTGAGGCTCTCTA–3′	280 bp

**Table 5:** Amplification conditions and reagents used in PCR for the study of the TF polymorphism.

Polymorphism	Amplification Conditions	Reagents
***TF* IVS 11 G>A (rs3811647)**	**1 cycle** Initial denaturation: 94 °C, 4 min **30 cycles** Denaturation: 94 °C, 30 s Annealing: 59 °C, 30 s Extension: 72 °C, 30 s **1 cycle** Final extension: 72 °C, 5 min	DNA [25 ng/μL]: 0.5 µL Primer Forward (25 pmol): 0.5 μL Primer Reverse (25 pmol): 0.5 μL * B Buffer (10×): 23.4 μL GoTaq G2 Flexi DNA polymerase (5 U/μL): 0.1 μL
* B Buffer: 50 mM KCl_2_; 10 mM Tris-HCl pH = 8.8; 150 μM MgCl_2_; 0.01%(*p*/*v*) gelatine, 4 × 25 mM dNTPs		

**Table 6:** Primers used and amplification fragments obtained in long-PCRs of *HAMP* promoter, *BMP2* exon 3 and *SLC40A1* fragments 1, 2, and 3.

Polymorphism	Primers	Amplification Fragment
***HAMP*-1010 C>T (rs10414846)** ***HAMP*-582 A>G (rs10421768)**	Forward: 5′–ACTGAGAAGGCAGCCCCTG–3′ Reverse: 5’–CGTGCCGTCTGTCTGGC–3′	1139 bp
***BMP2* 570 A>T (rs235768)**	Forward: 5′–ACAGAGAGAAGGGAGGCTCC3–3′ Reverse: 5’–CGACACCCACAACCCTCCAC–3′	1031 bp
***SLC40A1* frag. 1**	Forward: 5′–ACCTGCTGAGCCTCCCAAA–3′ Reverse: 5’–ACAACTGGCTAGAACGAAAGGAAATAAA–3′	991 bp
***SLC40A1* frag. 2**	Forward: 5′–TCCTGAGTACAATAGACTAGAAACGAAAAATA–3′ Reverse: 5′–TTACAGCCTCATTTATCACCACCGATT–3′	3747 bp
***SLC40A1* frag. 3**	Forward: 5′–TGAGGCAAATTTAGTGGGACTTGACC–3′ Reverse: 5′–GGGGAATTCAGTGTTATCATTATAGTCTC–3′	4175 bp

**Table 7:** Amplification conditions and reagents for long-PCRs of *HAMP* promoter, *BMP2* exon 3 and *SLC40A1* fragments 1, 2, and 3.

Polymorphism	Amplification Conditions	Reagents
***HAMP* -1010 C>T (rs10414846)** ***HAMP* -582 A>G (rs10421768)**	**1 cycle** Initial denaturation: 95 °C, 10 min **30 cycles** Denaturation: 95 °C, 45s Annealing: 67 °C, 45 s Extension: 72 °C, 100 s **1 cycle** Final extension: 72 °C, 10 min	DNA [25 ng/μL]: 0.5 µL Primer Forward (25 pmol/μL): 0.5 μL Primer Reverse (25 pmol/μL): 0.5 μL * B Buffer (10x): 23.4 μL GoTaq G2 Flexi DNA polymerase (5 U/ μL): 0.1 μL
***BMP2* 570 A>T (rs235768)**	**1 cycle** Initial denaturation: 95 °C, 10 min **30 cycles** Denaturation: 95 °C, 45 s Annealing: 62 °C, 45s Extension: 72 °C, 80 s **1 cycle** Final extension: 72 °C, 10 min	DNA [25 ng/μL]: 0.5 µL Primer Forward (25 pmol/μL): 0.5 μL Primer Reverse (25 pmol/μL): 0.5 μL * B Buffer (10x): 23.4 μL GoTaq G2 Flexi DNA polymerase (5 U/μL): 0.1 μL
***SLC40A1* frag. 1**	**1 cycle** Initial denaturation: 95 °C, 4 min **30 cycles** Denaturation: 95 °C, 30 s Annealing: 69 °C, 30s Extension: 72 °C, 1 min **1 cycle** Final extension: 72 °C, 10 min	DNA [25 ng/μL]: 2 µL Primer Forward (25 pmol/μL): 0.5 μL Primer Reverse (25 pmol/μL): 0.5 μL Premix Ex Taq Hot Start (Takara): 12.5 μL H_2_O: 9.5 μL
***SLC40A1* frag. 2**	**1 cycle** Initial denaturation: 95 °C, 4 min **30 cycles** Denaturation: 95 °C, 45s Annealing: 62 °C, 45s Extension: 72 °C, 80s **1 cycle** Final extension: 72 °C, 10 min	DNA [25 ng/μL]: 1 µL Primer Forward (25 pmol/μL): 0.5 μL Primer Reverse (25 pmol/μL): 0.5 μL Premix Ex Taq Hot Start (Takara): 12.5 μL H_2_O: 10.5 μL
***SLC40A1* frag. 3**	**1 cycle** Initial denaturation: 95 °C, 4 min **30 cycles** Denaturation: 95 °C, 45 s Annealing: 60 °C, 30s Extension: 72 °C, 5 min **1 cycle** Final extension: 72 °C, 10 min	DNA [25 ng/μL]: 1 µL Primer Forward (25 pmol/μL): 0.5 μL Primer Reverse (25 pmol/μL): 0.5 μL Premix Ex Taq Hot Start (Takara): 12.5 μL H_2_O: 10.5 μL
* B Buffer: 50 mM KCl_2_; 10 mM Tris-HCl pH = 8.8; 150 μM MgCl_2_; 0.01%(*p*/*v*) gelatine, 4 × 25 mM dNTPs		

**Table 8:** Association of baseline metabolic/cellular parameters with fibrosis stage before DAAs treatment.

Baseline Parameter	F_1/2 (before DAAs treatment)_		F_3/4 (before DAAs treatment)_		*p* Value *
	Mean	95% CI	Mean	95% CI	
Fe (μg/dL)	115.42	[108.47–122.38]	135.67	[123.56–147.78]	0.006
TS (%)	39.56	[34.72–44.40]	41.66	[37.27–46.04]	0.258
TIBC (μg/dL)	310.79	[300.40–321.17]	335.31	[320.09–350.54]	0.004
Ft (ng/mL)	189.89	[167.58–212.19]	336.98	[259.04–414.92]	0.002
* Mann–Whitney Test; 95% CI—95% confidence interval for mean					

**Table 9:** Association of *SCL40A1* polymorphisms with fibrosis stage before DAAs treatment.

Polymorphism	Genotype	F_1/2_ _(Before DAAs Treatment)_		F_3/4_ _(Before DAAs Treatment)_		*p* Value	OR	CI 95%
		*n*	%	*n*	%			
***SLC40A1*** **(rs1439816)**	GG	36	53.73	32	82.05			
	GC	26	38.81	5	12.82	**0.011** **	NA	NA
	CC	5	7.46	2	5.13			
***SLC40A1*** **(rs1439816)**	GG	36	53.73	32	82.05	**0.004** *	3.937	[1.525–10.162]
	GC or CC	31	46.27	7	17.95		1	-
* Fisher exact test; ** Pearson chi-square test; OR—odds ratio; CI 95–95% confidence interval; NA—not applicable; For dominant and recessive models, only significant results are shown								

**Table 10:** Association of *SLC40A1* polymorphisms with the regression of liver fibrosis from F4 (cirrhosis) to F1, 2, or 3 after DAAs treatment.

Polymorphism	Genotype	F_4 (Before DAAs Treatment)_ to F_1/2/3 (After DAAs Treatment)_		F_4 (Before DAAs Treatment)_ to F_4 (After DAAs Treatment)_		*p* Value	OR	CI 95%
		*n*	%	*n*	%			
***SCL40A1*** **(rs11568351)**	GG	10	90.91	7	46.67	0.036 *	1	[1.155–113.115]
	GC	1	9.09	8	53.33		11.429	
* Fisher exact test; OR—odds ratio; CI 95—95% confidence interval								

**Table 11:** Association of genetic polymorphisms with metabolic parameters of iron metabolism before DAAs treatment.

Polymorphism	Genotype	Fe (mg/dL)			TIBC (mg/dL)			Ft (ng/mL)		
		Mean	95% CI	*p* Value	Mean	95% CI	*p* Value *	Mean	95% CI	*p* Value
***HFE*** **C282Y** **(rs1800562)**	CC	120.95	[113.34–128.56]	0.009 *	321.27	[311.57–330.97]	0.268 *	229.46	[189.95–268.98]	0.005 *
	CY	157.18	[125.46–188.90]		301.82	[270.75–332.89]		502.27	[260.55–744.00]	
***TF *IVS 11 G>A** **(rs3811647)**	GG	123.04	[109.98–136.10]		307.22	[293.37–321.07]		240.45	[186.40–294.50]	
	GA	124.11	[105.39–142.82]	0.445 **	334.29	[309.00–359.57]	0.038 **	265.29	[201.29–329.29]	0.770 **
	AA	141.14	[108.27–174.01]		329.43	[277.38–381.48]		233.14	[127.73–338.58]	
	GG	-	-	-	307.22	[293.37–321.07]	0.011 *	-	-	-
	GA or AA	-	-		333.48	[311.53–355.42]		-	-	
***BMP2* 570 A>T** **(rs235768)**	AA	-	-	-	-	-	-	146.80	[54.86–238.74]	0.033 *
	AT or TT	-	-		-	-		273.46	[232.73–314.19]	
* Mann–Whitney Test; ** Kruskal–Wallis Test; 95% CI—95% confidence interval for mean; For dominant and recessive models, only significant results are shown										

## Data Availability

The datasets used and analyzed during the current study are available from the corresponding author upon reasonable request.
